# Long-Term Hemorrhage and Reperfusion Rates of Coiled Aneurysms: A Single-Center Experience

**DOI:** 10.3390/jcm13175223

**Published:** 2024-09-03

**Authors:** Lukas Andereggen, Salome L. Bosshart, Serge Marbacher, Basil E. Grüter, Jatta Berberat, Gerrit A. Schubert, Javier Anon, Michael Diepers, Hans-Jakob Steiger, Luca Remonda, Philipp Gruber

**Affiliations:** 1 Department of Neurosurgery, Kantonsspital Aarau, 5001 Aarau, Switzerland; lukas.andereggen@gmail.com (L.A.); gerrit.schubert@ksa.ch (G.A.S.); hsteiger@uni-duesseldorf.de (H.-J.S.); 2 Faculty of Medicine, University of Bern, 3012 Bern, Switzerland; luca.remonda@bluewin.ch; 3 Department of Neurosciences, University of Calgary, Calgary, AB T2N 1N4, Canada; 4 Faculty of Medicine, University of Zürich, 8006 Zürich, Switzerland; basil.grueter@ksa.ch; 5 Department of Neuroradiology, Kantonsspital Aarau, 5001 Aarau, Switzerland; jatta.berberat@ksa.ch (J.B.); javier.anon@ksa.ch (J.A.); michael.diepers@ksa.ch (M.D.); 6 Department of Neurosurgery, RWTH Aachen University, 52062 Aachen, Germany

**Keywords:** aneurysms, endovascular coil embolization, reperfusion, hemorrhage

## Abstract

**Background:** The endovascular approach has emerged as standard therapy for many intracranial aneurysms (IAs) to prevent hemorrhage, yet its long-term durability varies considerably. The aim of this study was to evaluate the safety and effectiveness of an initially deliberate endovascular approach regarding IA hemorrhage rates over a long-term follow-up period. **Methods:** This retrospective single-center study included all consecutive patients with endovascularly treated IAs who presented between January 2008 and December 2020 with a follow-up of at least 12 months. The primary endpoint was the proportion of patients with long-term IA hemorrhage rates and reperfusion. The secondary endpoint was treatment-related morbidity and mortality. Independent risk factors for IA reperfusion over the long term were analyzed using multivariate logistic regression. **Results:** Endovascular treatment was the therapy of choice for 333 patients with IAs, among whom 188 (57%) experienced rupture upon presentation. Complete coiling (Raymond I) was noted in 162 (49%) of the patients, with primary supportive devices being used in 51 (15%) patients. After a median (±SD) follow-up time of 34 ± 41 months (range 12–265 months), IA reperfusion was noted in 158 (47%), necessitating retreatment in 105 (32%) of the patients. Over the long term, hemorrhage was noted in four (1%) patients. Multivariate analysis revealed aneurysmal multilobarity (HR 1.8, 95%CI 1.2–2.7; *p* = 0.004) and a patient age of ≥50 years (HR 1.7, 95% CI 1.1–2.5, *p* = 0.01) as independent predictors of reperfusion over time. Intervention-related morbidity was noted in 16 (4.8%) patients, namely, thrombosis formation and contrast extravasation in 8 (2.4%) patients each, while no intervention-induced mortality was observed. **Conclusion:** In the long term, the hemorrhage rate in patients with IA with an initially more conservative endovascular approach is low. Therefore, a deliberate endovascular treatment approach might be justified.

## 1. Introduction

The treatment strategy for intracranial aneurysms (IAs) varies considerably, with endovascular coil embolization being increasingly considered as the primary treatment option [1,2]. The main aim of IA treatment is the prevention of rebleeding in ruptured IAs and the prevention of hemorrhage in unruptured IAs [3]. However, IA recurrence after treatment with endovascular coiling is not infrequent and is reported to be as high as 33.6%, and long-term durability remains elusive [4,5,6]. Several risk factors for the recurrence of IAs have been described, such as age; sex, aneurysm location (i.e., posterior circulation or MCA location); aneurysmal wall enhancement (AWE) in large, coiled, unruptured IAs; and a low volume–embolization ratio [7,8]. Among these factors, some are clearly linked to IA recurrence, such as the aneurysmal neck configuration (i.e., wide neck) and the size of the aneurysms (i.e., large IA), whereas others, such as the rupture status, smoking, and other modifiable factors (e.g., blood pressure and diabetes), are less clearly associated [9]. The underlying pathological mechanisms of IA recurrence are aneurysmal growth, coil compaction, and coil migration through the aneurysm wall [10]. Of those, coil compaction might be the most important factor for IA recurrence due to the dissolution of the intra-aneurysmal thrombus and the water-hammer effect [11].

The significance of these factors regarding hemorrhage rates in the long term is ambiguous. For instance, the CARAT study showed that, for ruptured IAs, the larger the IA remnant, the higher the risk of rehemorrhage [12]. In addition, the treatment of recurrent IAs seems to be associated with greater periprocedural risks [13]. Thus, to reduce the risk of hemorrhage, prompt detection and retreatment are important.

Meanwhile, several techniques, such as balloon-assisted (BAC) or stent-assisted coiling (SAC), as well as novel devices, such as flow diverters and intrasaccular devices (e.g., Woven EndoBridge (WEB, Microvention, Terumo, CA, USA) and Contour (Stryker Neurovascular, Kalamazoo, MI, USA), have been introduced over the last several decades to lower the hemorrhage and reperfusion rates.

The aim of this study was to evaluate the safety and effectiveness of a deliberate endovascular approach regarding IA reperfusion and hemorrhage rates over a long-term follow-up period and the associated morbidity.

## 2. Materials and Methods

### 2.1. Patient Inclusion Criteria

Patients with ruptured and unruptured IAs referred to a single tertiary care hospital between January 2008 and December 2020 were included (*n* = 810; catchment area of around 800,000). Of these, 422 patients were treated with an endovascular approach. We included all patients who underwent primarily conventional coil embolization with or without supportive devices, and a follow-up of at least 12 months was required to document the risk of clinical and aneurysm-related adverse events. In brief, our specific treatment criteria (endovascular vs. microsurgery) were the following: small-necked (<4 mm, dome–neck ratio < 2) aneurysms with an anatomical midline distribution were favored for coiling, whereas wide-necked aneurysms and aneurysms located at the middle cerebral artery (MCA)–M1 bifurcation were favored for a microsurgical treatment.

Patients with fusiform or dissecting aneurysms and those with aneurysms needing another primarily endovascular intervention (e.g., a flow diverter or parent vessel occlusion (PAO)) strategy were excluded from this study. Patients with aneurysmal subarachnoid hemorrhage (aSAH)-related death within <6 months of study inclusion were censored for the long-term analysis [14]. Finally, 333 patients met these criteria. This study was approved by the local ethics committee (EKNZ Nr. 2020-02249), and the need for informed consent was waived.

### 2.2. Study Endpoints

The primary endpoint was the proportion of patients with long-term IA hemorrhage and reperfusion rates. The secondary endpoint was treatment-related morbidity and mortality.

### 2.3. Assessment of Risk Factors for IA Recurrence

The following clinical and radiological risk factors were entered into the database for the assessment of the risk of IA reperfusion: patient age, sex, presence of aSAH at baseline, multiple IAs, IA multilobarity, baseline aneurysm neck diameter (“neck size”), IA location, the primary use of supportive devices, and completeness of initial coil embolization (modified Raymond–Roy occlusion classification, mRROC).

### 2.4. Neurointerventional Procedure

Cerebral digital subtraction angiography (DSA) was performed under general anesthesia using a biplane Angio system (Allura Xper FD20/20, Philips, Amsterdam, The Netherlands). Coil embolization was usually performed using a femoral approach. After placing a 6F or 7F guide catheter—for most of the cases—an Excelsior-SL 10 microcatheter (Stryker Neurovascular, Kalamazoo, MI, USA) was used. Detachable bare metal coils—for most of the cases, complex target coils (Stryker Neurovascular, MI, USA)—were used, and if needed, a stent or balloon was placed to allow for secured coil detachment (i.e., supportive devices). In cases of stent placement, 300 mg of aspirin was administered intravenously before stent deployment and coiling of the aneurysm, and 75 mg/day of clopidogrel was added after intervention depending on the clinical course after aSAH. In unruptured IAs, patients were preloaded with aspirin (100 mg/day) and clopidogrel (75 mg/day) three to five days prior to the intervention.

### 2.5. Procedural Complications

Peri-interventional complications such as aneurysm rupture, thromboembolic events, vessel dissections, and/or vasospasms with subsequent neurological deficits were recorded.

### 2.6. Clinical and Angiographic Follow-Up

Angiographic status and follow-up results, including IA reperfusion, retreatment rates, and the use of supportive devices (i.e., balloon- or stent-assisted coiling), were recorded from the time of study entry to the last follow-up. For the radiological follow-up, our patients usually received a first follow-up DSA and MRI with TOF (time-of-flight) angiography 6 months after the intervention. If not otherwise specified, the second follow-up was performed 2 years after the intervention using MRI with TOF, which is a reliable and less invasive alternative to DSA [15,16]. Thereafter, the follow-up periods were steadily increased depending on the follow-up results. The decision to recommend retreatment, as well as that of the approach to retreatment (endovascular vs. clipping), was made by a weekly multidisciplinary neurovascular board. If endovascular treatment was favored over the open surgical approach, the decision was based on favorable anatomical features of the aneurysm, as well as a low associated risk of endovascular retreatment in recurrent IAs as determined by factors such as the neck size, location of the aneurysm, age of the patient, and comorbidities [17,18].

### 2.7. Statistical Analysis

The data were analyzed using IBM SPSS statistical software version 24.0 (IBM Corp., New York, NY, USA) and are presented with descriptive statistics as means ± SD for continuous variables and as counts and frequencies for categorical variables. Student’s t-test was used for normally distributed data, and the Mann–Whitney test was used for skewed or non-normal data. A statistical analysis of categorical variables was carried out with the χ^2^ test or Fisher’s exact test, as appropriate.

The association of the primary endpoint with potential independent risk factors was assessed by performing a time-dependent multivariable regression analysis to calculate hazard ratios (HRs). The following baseline risk factors were assessed: age ≥ 50 years, sex, presence of aSAH, multiple aneurysms, multilobarity, and the use of a supportive device at baseline. The multivariable Cox regression analysis included all dependent risk factors in the univariable regression with a *p*-value of <0.3 *p*-values that were ≤0.05 were considered statistically significant.

## 3. Results

### 3.1. Patient and IA Characteristics

The patient demographics, IA characteristics, and treatment approaches are summarized in Table 1. A total of 227 (68%) women and 106 (32%) men met the inclusion criteria. The mean age at diagnosis was 56 ± 13 years. Vascular risk factors, including active smoking status in 33% of patients (110/333), hypertension in 48% of patients (160/333), diabetes in 8% of patients (27/333), and obesity (i.e., BMI ≥ 30 kg/m^2^) in 18% of patients (58/333), were noted. Alcohol and/or cocaine abuse was recorded in 4% and 1% of patients (14/333 and 3/333), respectively. A total of 57% of patients (188/333) presented with aSAH due to IA rupture at baseline.

Fifty-one (15%) IAs were treated primarily with a supporting device (i.e., stent- or balloon-assisted coil embolization). Initial complete occlusion (mRROC I) was reported in 49% of patients (162/333), near-complete occlusion (mRROC II) was reported in 33% of patients (111/333), and incomplete occlusion (mRROC III) was reported in 18% of patients (60/333). In the mRROC III group, 56% of patients (34/60) had mRROC IIIa, whereas 44% of patients (26/60) had mRROC IIIb. The IA neck size (*p* = 0.01) was significantly larger and multilobarity (*p* = 0.01) was significantly more common in patients with IA recurrence in the long term, as was their retreatment rate (*p* < 0.001).

### 3.2. Risk Factors for IA Recurrence

After a median (±SD) follow-up time of 34 ± 41 months (range, 12–265 months), IA reperfusion was noted in 47% of patients (158/333), and retreatment was necessary in 32% of patients (105/333). Aneurysmal multilobarity (HR 1.8, 95%CI 1.2–2.7; *p* = 0.004) and a patient age of ≥50 years (HR 1.7, 95% CI 1.1–2.5, *p* = 0.01) were independent risk factors for IA reperfusion over the study period (Table 2), but IA size (large aneurysms > 7 mm, *p* = 0.33) and IA neck size (*p* = 0.32) were not.

### 3.3. IA Hemorrhage Rates

IA hemorrhage was noted in 1.2% of patients (4/333), and, in 0.9% of patients (3/333), IA reperfusion over time was noted. The relative risk (RR) for hemorrhage in patients with reperfused IAs was 1.01. (95% CI 1.0–1.1). The functional outcome at the last follow-up was not significantly different in patients with or without hemorrhage (mRS ≤ 2 in 75% vs. 82% of patients, *p* = 0.56), but it was in patients with initial aSAH (mRS ≤ 2 in 72% vs. 94% of patients, *p* < 0.001).

### 3.4. Treatment-Related Morbidity and Mortality

Intervention-related morbidity was noted in 4.8% of patients (16/333), namely, thrombosis formation and contrast extravasation in 2.4% of patients (8/333) each, and temporary cerebral vasospasm occurred in 1.2% of patients (4/333); no intervention-induced mortality was observed.

## 4. Discussion

The main findings of this study can be summarized as follows: (1) The rate of IA hemorrhage was low (1.2%) despite there being a reperfusion rate of 47% over time. (2) IA multilobarity was an independent risk factor for reperfusion, which may justify a more aggressive treatment approach in such cases. However, a deliberate treatment approach is both safe and functionally effective.

Endovascular coil embolization has become the treatment of choice for many IAs to prevent aSAH and its associated high mortality and long-term morbidity [19]. Although the risk of coil embolization is minimal for most aneurysms [20]. IA recurrences treated with endovascular coiling remain arduous [7]. Different IA characteristics, technical approaches, and clinical factors have been found to be associated with increased rates of IA reperfusion [21,22,23]. Incomplete initial coil embolization or low coil-packing density [24,25]; IA location (i.e., posterior or MCA aneurysms) [26,27,28]; a previous history of aSAH [21,22]; and large, wide-neck IAs [21,28], as well as AWE in large coiled unruptured IAs [8], have been linked to a higher probability of reperfusion. In particular, the configuration of the vascular structures may contribute to aneurysm growth and reperfusion [29,30,31,32].

Interestingly, in contrast to the wide-necked IAs, early major recurrence has been described for small-neck IAs instead [33]. In line with these findings, we noted significantly higher retreatment rates in recurrent and wide-neck aneurysms, with IA multilobarity being an independent risk factor for IA recurrence. While a multilobular shape is strongly associated with IA rupture states [34,35,36], an irregular IA shape is likewise associated with IA recanalization [37,38], corroborating our results. These IAs remain difficult to treat on an endovascular basis, with a recent study confirming that, even with novel techniques such as WEB devices for the treatment of wide-neck IAs, an irregular shape of the angiographic aneurysm remnant is an independent risk factor [38].

Nevertheless, considering our study’s results, a more aggressive approach might be justified, while a more deliberate treatment approach can be both safe and functionally effective regarding the low rebleeding rates in most IAs. Namely, we noted high recanalization and retreatment rates in 47% and 32% of patients, respectively.

However, novel endovascular devices, such as flow diverters and intrasaccular flow-disruptors (e.g., WEB, Contour), have been reported to have lower recurrence and retreatment rates than those of conventional coiling techniques. A recent meta-analysis of 11 studies on flow diverters showed a long-term retreatment rate of 5% [39]. Comparable findings were reported in a meta-analysis of 15 studies of WEB devices, showing a retreatment rate of around 6% with overall low morbidity and mortality rates [40]. However, more recent data from large WEB series indicated retreatment rates of 8.8% and 12.3%, respectively, but these values are still lower than those of conventional coil embolization [41,42]. A comparative study of another flow disruptor, Contour—specifically designed for wide-neck bifurcational aneurysms—vs. coil embolization revealed a lower retreatment rate (7% vs. 21%) [43]. Thus, these endovascular devices might be an alternative to conventional coil embolization.

The associated low morbidity and low risk of (re-)hemorrhage (1.2%) might justify a more conservative approach. These findings are in line with the previously reported risk of rehemorrhage as ranging between 0.6% and 2.6% [44]. As for aneurysm treatments, simple coil embolization is currently the most common, primary procedure [45]. Indeed, IA recanalization with a deliberate coil embolization approach that does not strictly use supporting devices was a risk factor in the univariable analysis (*p* = 0.04) but not in the multivariable analysis. This is in line with results showing a significantly lower recurrence rate of IA reperfusion when using stent-assisted coiling [46]. This is not an uncommon finding given that IA treatments using supporting devices present a minimal risk of recanalization once occlusion is achieved [47]. Higher rates of recanalization with coiling have been reported before [48], particularly in those with posterior circulation [49]. Interestingly, however, posterior circulation has not been shown to be an independent risk factor for aneurysm growth [32]. In our cohort, a posterior IA location was not associated with higher rates of reperfusion, nor was it a risk factor for aneurysm growth.

Our findings enrich the understanding of the recurrence of IAs using a more conservative and deliberate primary treatment approach with coil embolization, and, considering the low rebleeding rates, it might be justified. Nevertheless, for patients with IA shape irregularities, which are an independent risk factor, it will become crucial to apply novel endovascular devices in the treatment of these complex IAs and attenuate their recurrence.

## 5. Study Limitations

This study suffers from the limitations of every retrospective study, as well as its single-center design. The relatively low number of aneurysms treated at our center per year and, therefore, the relatively small sample size affects the power and significance of the findings and limit the ability to perform a detailed statistical analysis of rebleeding risk.

In addition, there have been tremendous advances in material and technical treatment options over the last several decades.

## 6. Conclusions

In the long term, the hemorrhage rate in patients with IA with an initially more conservative endovascular approach is low. Therefore, a deliberate endovascular treatment approach might be justified.

## Figures and Tables

**Table 1:** Patient demographics and aneurysm characteristics.

Characteristics	No IA Reperfusion	IA Reperfusion	All Patients	*p*-Value
Total cases, *n* (%)	175 (53)	158 (47)	333 (100)	
Age (mean ± SD), years	56 ± 14	55 ± 13	56 ± 13	0.61
Female sex, *n* (%)	117 (67)	110 (70)	227 (68)	
Hypertension, *n* (%)	82 (47)	78 (49)	160 (48)	0.74
Diabetes, *n* (%)	9 (5)	18 (11)	27 (8)	0.05
Obesity, *n* (%)	25 (14)	33 (21)	58 (18)	0.15
Active smoking, *n* (%)	62 (36)	48 (31)	110 (33)	0.04
Alcohol abuse, *n* (%)	7 (4)	7 (4)	14 (4)	1
Cocaine abuse, *n* (%)	0 (0)	3 (2)	3 (1)	0.11
Posterior circulation of IAs, *n* (%)	50 (29)	54 (34)	104 (31)	0.29
Neck size (median, IQR)	2.4 (1–3.7)	3 (2.3–4)	2.8 (2–3.7)	0.01
Multiple IAs, *n* (%)	66 (38)	50 (32)	116 (35)	0.25
Multilobarity of IAs, *n* (%)	84 (63)	60 (47)	144 (55)	0.01
IA rupture (i.e., aSAH), *n* (%)	108 (62)	80 (51)	188 (57)	0.05
IA coiling complete, *n* (%)	88 (50)	74 (47)	162 (49)	0.58
Supportive devices used, *n* (%)	28 (16)	23 (15)	51 (15)	0.76
IA retreatment over time, *n* (%)	14 (8)	91 (58)	105 (32)	<0.001
IA hemorrhage over time, *n* (%)	1 (0.6)	3 (1.9)	4 (1.2)	0.35

Abbreviations: *n*, number; IQR, interquartile range; IA, intracranial aneurysm.

**Table 2:** Clinical and radiological predictors (hazard ratio with 95% CI) for IA reperfusion.

Risk Factors for Reperfusion	Univariable Analysis of HR (95% CI)	*p*-Value	Multivariable Analysis of HR (95% CI)	*p*-Value
Age > 50 years	1.6 (1.1–2.2)	0.01	1.7 (1.1–2.5)	0.01
Sex (i.e., women)	1.1 (0.8–1.5)	0.64		
aSAH at baseline	1.2 (0.9–1.7)	0.2	1.0 (0.6–1.4)	0.80
Posterior circulation of IA	1.0 (0.7–1.5)	0.81		
IA size (i.e., large IA)	1.9 (0.5–6.7)	0.33		
Neck size of IA	1.1 (0.9–1.2)	0.32		
Multiple aneurysms	1.3 (0.9–1.9)	0.11	1.3 (0.9–2.0)	0.22
Multilobarity of IA	1.7 (1.2–2.5)	0.004	1.8 (1.2–2.7)	0.004
Initial complete IA coiling	1.0 (0.7–1.4)	0.91		
Supportive devices used	0.6 (0.4–1.0)	0.04	0.7 (0.4–1.3)	0.27

Abbreviations: *n*, number; IQR, interquartile range; IA, intracranial aneurysm; HR, hazard ratio; CI, confidence interval.

## Data Availability

The authors agree to share data upon request.
